# What are patients’ and healthcare professionals’ views on managing penicillin allergy? A qualitative evidence synthesis

**DOI:** 10.1093/jacamr/dlag049

**Published:** 2026-04-20

**Authors:** Kornelija Kildonaviciute, Marta Wanat, Nia Wyn Roberts, Jonathan Sandoe, Sarah Tonkin-Crine

**Affiliations:** Nuffield Department of Primary Care Health Sciences, University of Oxford, Oxford OX2 6GG, UK; Nuffield Department of Primary Care Health Sciences, University of Oxford, Oxford OX2 6GG, UK; National Institute for Health and Care Research Health Protection Research Unit in Healthcare Associated Infections and Antimicrobial Resistance, University of Oxford, Oxford, UK; Bodleian Health Care Libraries, University of Oxford, Oxford, UK; Leeds Institute of Medical Research, School of Medicine, University of Leeds, and Leeds Teaching Hospitals NHS Trust, Leeds, UK; Nuffield Department of Primary Care Health Sciences, University of Oxford, Oxford OX2 6GG, UK; National Institute for Health and Care Research Health Protection Research Unit in Healthcare Associated Infections and Antimicrobial Resistance, University of Oxford, Oxford, UK

## Abstract

**Objectives:**

To review the existing literature on patients’ and healthcare professionals’ views on managing penicillin allergy and provide an interpretation of the evidence that will inform the development of strategies to increase penicillin allergy evaluation and testing.

**Methods:**

We systematically searched six databases for qualitative or mixed-methods studies reporting patients’ and healthcare professionals’ views on managing penicillin allergy. We followed three stages of thematic synthesis, including line-by-line coding, development of descriptive themes, and development of analytical themes, to analyse and interpret the findings of eligible studies.

**Results:**

We included 21 papers in the review and using thematic synthesis developed five analytical themes: (i) investigation of penicillin allergy was not a priority; (ii) healthcare systems did not support penicillin allergy assessment; (iii) penicillin allergy assessment required specific training; (iv) uncertainty over responsibility in managing penicillin allergy; and (v) management of penicillin allergy was associated with perception of risk and diagnostic uncertainty.

**Conclusions:**

To increase penicillin allergy assessment and testing, the lack of prioritization of penicillin allergy assessment in clinical practice should be addressed. Education of patients and healthcare professionals should emphasize the benefits of penicillin allergy assessment and explain the risks associated with allergy testing and subsequent use of penicillin. Healthcare systems–related barriers hindering penicillin allergy assessment could be reduced through the redesign of electronic medical records and the revision of antibiotic guidelines. Future research should explore novel models for penicillin allergy assessment services involving different healthcare settings.

## Introduction

The discovery of penicillin paved the way for the development of modern-day antibiotics and undisputably transformed the treatment of infections.^1,2^ However, since its use in human trials, penicillin has been associated with adverse drug reactions.^3^ Although penicillin-associated anaphylaxis and other immunologically mediated hypersensitivity reactions are extremely rare,^4^ to date, penicillin allergy remains among the most reported drug allergies, with an estimated global prevalence of 9.4%.^5^

Having a documented penicillin allergy in medical records is associated with increased consumption of non-penicillin antibiotics,^6,7^ poses a risk of adverse health outcomes^8,9^ and increases healthcare costs.^10^ Considering that over 90% of penicillin allergy records have been found to be incorrect after assessment and testing,^11^ millions of people could be at unnecessary risk of adverse health outcomes. Therefore, assessment of penicillin allergy has become part of the global antimicrobial stewardship agenda.^12,13^

Penicillin allergy assessment is complex and is traditionally performed by allergy specialists.^14^ However, due to limited availability of allergy specialists,^15^ research efforts in managing penicillin allergy have been redirected to focus on the development of penicillin allergy assessment pathways outside the specialist setting to tackle the burden of unconfirmed penicillin allergy records.^16^ In addition to safety and efficacy considerations, understanding the views of patients and healthcare professionals concerning the new penicillin allergy assessment pathways was identified as a key prerequisite for a successful uptake of these pathways in clinical practice.^17^

In this qualitative evidence synthesis, we aimed to systematically review the existing literature on patients’ and healthcare professionals’ views on managing penicillin allergy in any healthcare setting and provide an interpretation of the evidence to inform the development of strategies to increase penicillin allergy assessment.

## Methods

The protocol for this review was registered and published with PROSPERO (CRD42024507644). The reporting of this review followed the Enhancing Transparency in Reporting the Synthesis of Qualitative Research (ENTREQ) framework.^18^ The reporting checklist is reported in Table S1 (available as Supplementary data at *JAC-AMR* Online).

### Defining the management of penicillin allergy

Informed by the published literature, we defined ‘management of penicillin allergy’ as a collection of behaviours conducted by either healthcare professionals or patients in relation to penicillin allergy, as described in Table 1.

**Table 1.** Summary of behaviours that encompass the ‘management of penicillin allergy’

Healthcare professionals’ behaviours	Patients’ behaviours
Taking an allergy history when an allergic reaction is suspected: the name of the penicillin thought to have caused the reaction (strength, formulation and route of administration), description of the reaction, indication for the penicillin being taken, the date and time of the reaction, the number of doses taken or number of days receiving the drug before onset of the reaction, and which drugs or drug classes to avoid in future^19^	Asking a healthcare professional if they have an allergy to penicillin after having an adverse reaction
Assessing the likelihood of a true allergy to penicillin using own professional judgement, protocols, guidelines and digital applications	N/A
Recording an allergy according to guidelines^19^	Carrying or using an allergy alert card
Prescribing an alternative antibiotic to a patient with a suspected penicillin allergy	Taking alternative antibiotics
Assessing patients’ medical needs and suitability for penicillin allergy testing	Seeking penicillin allergy assessment
Prescribing and administration of a direct oral challenge with penicillin for a suitable patient with a low-risk allergy history in a non-specialist setting^20^	Undergoing penicillin allergy assessment in a non-specialist setting
Referring a patient to a specialist for penicillin allergy assessment	Undergoing penicillin allergy assessment in a specialist setting
Making a diagnosis of no allergy or penicillin allergy based on the penicillin allergy assessment	N/A
Updating allergy records with the outcome of penicillin allergy assessment	Accepting the outcome of penicillin allergy assessment and/or renewed allergy status
Communicating the outcome of penicillin allergy assessment to patient and other healthcare professionals	Communicating the outcome of penicillin allergy assessment and/or renewed allergy status to healthcare professionals in future
Prescribing of penicillin-based antibiotics after a negative result of penicillin allergy assessment	Taking penicillin after a negative result of penicillin allergy assessment

N/A, not applicable.

### Search strategy

A comprehensive search strategy was developed with an information specialist (N.R.) to identify literature reporting patients’ and healthcare professionals’ views on managing penicillin allergy.

The electronic databases MEDLINE (OvidSP), CINAHL (EBSCOHost), Embase (OvidSP), PsycINFO (OvidSP), ASSIA (Proquest) and Google Scholar were searched using relevant title, abstract, author keywords and subject headings in April 2024 and November 2024. No date or language limits were applied. Forward citation, grey literature and website searches were also conducted. The full search strategy is provided in Table S2.

### Study selection

We included peer-reviewed qualitative or mixed-methods studies reporting qualitative findings of patients’ and/or healthcare professionals’ views on the predefined list of behaviours encompassing the management of penicillin allergy (Table 1). We set no limits for publication date, geographical location, participants’ age, healthcare professionals’ group or healthcare setting.

We excluded qualitative or mixed-methods studies that used surveys for data collection, systematic reviews, conference abstracts, posters and theses.

Papers were screened using Covidence software. K.K. screened all the papers at the abstract and full-text screening stage; M.W. screened 10% of papers at each stage. S.T.-C. vetted the papers where K.K. and M.W. disagreed over inclusion.

### Quality assessment

We used the Critical Appraisal Skills Programme’s Qualitative Checklist^21^ for quality assessment and considered the overall contribution of each paper to the research question. All papers were assessed independently by two reviewers. K.K., M.W., J.S. and S.T.-C. discussed the differences in quality assessment scores to resolve them. Final quality assessment scores are reported in Table S3.

### Data extraction

K.K. extracted the data of study characteristics using a form developed by the research team and imported full texts into NVivo version 14 for analysis. All text under the headings ‘results’, ‘findings’ and ‘discussion’ in the manuscripts was treated as data and was coded during synthesis.

### Thematic synthesis

We followed the three phases of thematic synthesis as described by Thomas and Harden:^22^ free line-by-line coding of the studies, organizing the codes into descriptive themes, and generation of analytical themes.

K.K. coded studies presenting combined views of patients and healthcare professionals first. S.T.-C., M.W. and J.S. reviewed the codes for consistency of interpretation. At this stage of data synthesis, we decided to specify the group of participants—e.g. nurses, doctors, parents, patients expressing a view on managing penicillin allergy—in the name of the code for clarity. Only where a particular view was shared by nurses, doctors, pharmacists or pharmacy technicians did we use the term ‘healthcare professionals’ in the name of the code.

K.K. then organized the codes to develop descriptive themes. After further discussion with S.T.-C., M.W. and J.S., K.K. revised the descriptive themes and coded remaining papers.

To generate analytical themes, K.K. mapped and interpreted links between the descriptive themes emerging from patients’ and healthcare professionals’ views on managing penicillin allergy. S.T.-C., M.W. and J.S. supported the development of analytical themes by offering comments and suggestions for revision. The list of analytical themes and associated descriptive themes is presented in Table S4. Individual study contribution to the development of analytical themes is mapped in Table S5.

## Results

We performed electronic searches on 4 April 2024 and 11 November 2024, which yielded 1818 studies. After the removal of duplicates and 25 animal studies, we screened 1178 abstracts and titles. We excluded 1029 papers and retrieved 149 full-text reports. At this stage we excluded 14 reports, which were conference proceedings. Following the full-text screening of 135 articles, 21 met the inclusion criteria.

**Figure 1.**
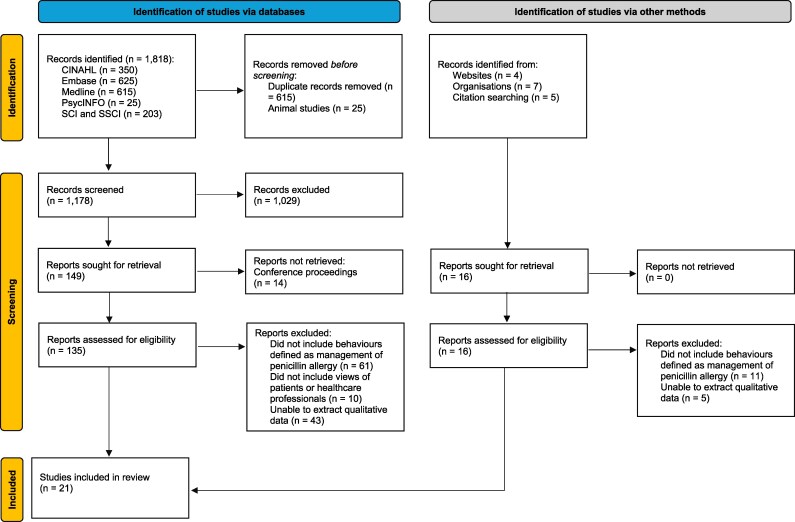
The PRISMA flowchart of the study selection process.

We identified 16 potential studies through citation search of articles selected for the review, grey literature and a website search, but none met the inclusion criteria. The PRISMA flowchart of the study selection process is presented in Figure 1. The summary of final quality assessment scores is presented in the Table S4. No papers were excluded based on quality. All studies were published since 2019.

Eight studies were conducted in the UK,^23–30^ six in the USA,^31–36^ three in the Netherlands,^37–39^ one in Canada,^40^ one in Norway,^41^ and two studies recruited participants from multiple countries.^42,43^ All studies, except Alqahtani *et al*.,^42^ were conducted in high-income countries.

Six studies were conducted in primary care,^24,25,27,32,36,37^ 11 in secondary care,^23,26,28–30,33–35,38,40,41^ and 3 recruited participants from both primary and secondary care.^39,42,43^ One study recruited participants in a community, outside a healthcare setting.^31^

Five studies explored the views of patients or their parents/carers,^23,28,31,33,40^ 10 studies explored the views of healthcare professionals,^26,30,34,35,37–39,41–43^ and 6 studies presented combined findings of patients’ and healthcare professionals’ views.^24,25,27,29,32,36^ In total, 215 patients (213 adults and 2 adolescents), 54 parents, 6 carers and 397 healthcare professionals participated in 21 studies.

In terms of professional representation, healthcare staff participating in the studies were general practiotioners (GPs)^24,25,27,37,39,43^ and general practice nurses;^27^ doctors, pharmacists and nurses based in a nursing home;^32^ doctors, nurses, pharmacists and pharmacy technicians based in an acute hospital;^26,29,30,34,35,38,39,41–43^ and non-clinical staff in a managerial or a leadership role in an acute hospital^29^ or a nursing home.^32^

Studies with patients recruited participants with a documented penicillin allergy who either had penicillin allergy assessment^23–25,27–29,33,40^ or did not.^31,32,36^

Characteristics of studies included in the review are summarized in Table 2.

**Table 2.** Characteristics of studies included in the review

Author	Title	Country	Context	Participants		Method of data collection	Method of data analysis	Research aims (as reported by authors)	Findings (as reported by authors)
**VIEWS OF PATIENTS and/or PARENTS or CAREGIVERS**									
Savic *et al.* (2019)^23^	Penicillin allergy delabelling ahead of elective surgery: feasibility and barriers	UK	A qualitative study conducted as part of a feasibility study using a penicillin allergy delabelling pathway including a direct oral challenge for elective surgical patients in a tertiary care setting in the UK	55 adults who were successfully delabelled		Follow-up phone consultation	Unspecified	To test the feasibility of incorporating a delabelling programme into an existing surgical care pathway; to assess the acceptability of this intervention among patients and clinicians, and the impact on prescribing during their surgery	Among the patients who were delabelled, the process was broadly considered to be ‘smooth’. Patients expressed low levels of anxiety about testing
Protudjer *et al.* (2020)^40^	GRAded oral challenge for drug allergy evaluation-delabelling described through families’ voices	Canada	A qualitative study conducted as part of a cohort study that assessed accuracy and negative predicted value of a graded penicillin provocation challenge in children with suspected allergy to amoxicillin referred to an allergy clinic in a children’s hospital in Canada	15 parents (14 individual parents, 1 mother-father dyad) of children who successfully passed a graded oral challenge as determined by a paediatric allergist		Semi-structured interviews	Thematic analysis; approach unspecified	To describe how families with a child previously labelled as antibiotic allergic, but who has subsequently been delabelled, perceive the experience of misdiagnosis and subsequent delabelling	Authors identified four themes: a red, raised rash results in a quick diagnosis despite lack of testing; sensitive care allays concerns; delabelling brings relief, but also mystery and calls for proper diagnosis; and quick diagnosis is reckless, but manageable through downward comparisons
Antoon *et al.* (2023)^33^	Parental perceptions of penicillin allergy risk stratification and delabelling	USA	A qualitative study conducted in a large academic children's hospital in the southern USA	2 adolescents and 21 parents; only 1 parent had a child who underwent and passed oral challenge during admission		Focus groups	Thematic analysis; inductive and deductive approaches	To evaluate parental and adolescent-patient perceptions of the penicillin allergy evaluation and delabelling process to better inform future efforts to implement hospital-based delabelling programmes	Parents’ and adolescents’ experiences with penicillin allergies consisted of four interconnecting themes: family context, the invitation to delabel, decision context, and penicillin allergy delabelling outcome Penicillin allergies remained a concern for families even if their children passed an oral challenge Some parents preferred testing to be performed in the hospital
Carter *et al.* (2023)^31^	Parent-reported penicillin allergies in children: a qualitative study	USA	A qualitative descriptive study with parents of children who have a reported penicillin allergy	18 parents of children with penicillin allergy (16 females and 2 males; 67% white; 83% non-Hispanic; 44% master’s degree)		Semi-structured interviews	Conventional content analysis	To describe parents’ experiences and perceptions regarding their child’s reported penicillin allergy and attitudes towards penicillin allergy testing with a goal to identify opportunities to engage parents in the recommended evaluation and management of their child's reported penicillin allergy	Parents were receptive to penicillin allergy testing for their child after learning the consequences of penicillin allergy and availability of allergy testing Four themes described included parents’ making sense of an allergy, parents’ impressions of allergy label, parents’ attitudes towards allergy testing, and parents’ desire to be informed of testing availability
Powell *et al.* (2024)^28^	Experiences of an inpatient penicillin allergy delabelling pathway: capturing the patient voice	UK	A qualitative study conducted as part of a feasibility study of an inpatient penicillin allergy delabelling pathway at a general district hospital without access to allergy service in England	19 adults (7 females, 12 males; age range 57–84; mean 73)		Semi-structured interviews	Thematic analysis, inductive and deductive approach	To explore the experiences of patients who have undergone penicillin allergy assessment with or without delabelling during hospital admission	Patients were often unable to recall the index reaction to penicillin, were unaware of the negative impact of penicillin allergy labels and reported that having penicillin allergy did not impact their care. Patients had different views on challenging their allergy status while they were acutely unwell. Patients declined testing because they felt they were at higher potential risk because they were older or had multiple comorbidities. Some patients felt they required a better explanation of the risks and benefits of delabelling
**VIEWS OF BOTH PATIENTS AND HEALTHCARE PROFESSIONALS**									
Wanat *et al.* (2019)^24^	Patient and primary care physician perceptions of penicillin allergy testing and subsequent use of penicillin-containing antibiotics: a qualitative study	UK	A qualitative study with patients with a penicillin allergy record and clinicians based in general practice in the UK	31 adults (25 women, 6 men; age range 17–72, mean 56); 16 experienced allergy testing, 4 tested positive and 11 tested negative for penicillin allergy; 1 inconclusive	19 GPs (16 women, 3 men; age range 34–60, mean age 42); 9 (47%) had experience in referring patients for penicillin allergy testing	Semi-structured interviews	Thematic analysis, inductive approach	To identify clinician and patient views and experiences of referring to or attending for penicillin allergy testing and the use of penicillin following a negative allergy test	Three themes captured patients’ views: personal relevance and benefits of the test; importance of safety and perceived risks of test; confidence in the test result Three themes captured clinicians’ views: doubts about removing penicillin allergy labels; knowledge of the allergy service and referral process; process of updating medical records
Wanat *et al.* (2021)^25^	Management of penicillin allergy in primary care: a qualitative study with patients and primary care physicians	UK	A qualitative study with patients with a penicillin allergy record and clinicians based in general practice in the UK	31 adults (25 women, 6 men; age range 17–72, mean 56); 16 experienced allergy testing, 4 tested positive and 11 tested negative for penicillin allergy; 1 inconclusive	19 GPs (16 women, 3 men; age range 34–60, mean age 42); 9 (47%) had experience in referring patients for penicillin allergy testing	Semi-structured interviews	Thematic analysis, inductive approach	To explore primary care physicians’ and patients’ views and understanding of penicillin allergy and their experiences of managing penicillin allergy in primary care	Patient-related themes included making sense of allergy, impact of allergy on managing health, and primary care physicians’ influence on patients’ perception of allergy Primary care physician–related themes included uncertainties around diagnosing penicillin allergy and making prescribing decisions for patients with penicillin allergy
Wanat *et al.* (2022)^27^	Mixed-methods evaluation of a behavioural intervention package to identify and amend incorrect penicillin allergy records in UK general practice	UK	A qualitative study conducted as part of a feasibility stage of a trial assessing intervention package centred around a penicillin allergy assessment pathway initiated in primary care involving 11 general practices based in northern England	10 adults (8 females, 2 males; age range 30–82, mean age 63.7); 8 tested negatively for penicillin allergy	7 primary care clinicians (4 females, 3 males; age range 43–57, mean age 46.4); 6 GPs, 1 advanced nurse practitioner	Semi-structured interviews	Thematic analysis, inductive approach	To investigate patients’ and clinicians’ perspectives on penicillin allergy testing as part of the mixed methods process evaluation of the ALABAMA feasibility trial	Clinicians were motivated by having access to penicillin allergy testing as they were convinced of the benefits of penicillin for their patients. Clinicians were often unsure about criteria for referral or what allergy testing involved, and reported little prior experience and knowledge in testing prior to the trial. Clinicians recognized the importance of updating allergy records following the test Patients were motivated to know if they were truly allergic but were concerned over the safety of penicillin allergy testing and therefore valued having an opportunity to address their concerns with their GP and hospital staff
Gillespie *et al.* (2023)^32^	Facilitators and barriers to verifying penicillin allergies in a veterans’ nursing home population	USA	A qualitative study assessing understanding and receptiveness to verification of penicillin allergies using a clinical pathway in a nursing home in the northeastern USA	9 participants (3 nursing home residents and 6 family members/healthcare proxies)	15 staff members (3 physicians, 5 nurses, 3 pharmacists, 4 hospital leadership)	Semi-structured interviews	Modified grounded theory approach	To identify potential facilitators and barriers to implementing a strategy to verify penicillin allergies and delabel low-risk nursing home patients	Staff and patient receptiveness and leadership support were the key facilitators, whilst family receptiveness and clinical resources were the main barriers to verifying penicillin allergies and delabelling low-risk nursing home patients Education and training and organizational policy were suggested as means to overcome these barriers
Ngassa *et al.* (2024)^36^	‘Let a sleeping dog lie’: perspectives from patients and clinicians about penicillin allergy delabelling	USA	A qualitative study conducted in an academic medical centre in the northeastern USA	21 patients	11 clinicians	Semi-structured interviews, in- person	Unspecified	To understand the barriers and facilitators for penicillin allergy delabelling	Barriers included patients’ and clinicians’ reluctance to discuss penicillin allergy, perceived abundance of alternative antibiotics, women from racial and ethnic minority backgrounds had concerns deterring interest in delabelling, clinicians concern about harms of delabelling and operational barriers. Facilitators included patients’ interest in delabelling, clinicians’ motivation to give the most appropriate antibiotic treatment and improving penicillin allergy delabelling discussions
Jani *et al.* (2024)^29^	Factors influencing implementation and adoption of direct oral penicillin challenge for allergy delabelling: a qualitative evaluation	UK	A qualitative study conducted as part of a feasibility study of a delivery of a direct penicillin challenge to inpatients and outpatients at three acute care hospitals in England	43 adults (20 females, 23 males; 77% white British); 40 agreed and 3 declined direct oral challenge	28 (16 females, 12 males; 47% white, 29% Asian or Asian British, 4% Arabic, 21% did not state)	Semi-structured interviews with patients and focus groups with healthcare professionals	Thematic analysis using inductive and deductive approaches	To explore the behaviour, attitudes and acceptability of patients, healthcare professionals and managers regarding the use of direct oral challenge to remove penicillin allergy labels in low-risk patients	Knowledge, beliefs about capabilities and consequences, resources, environmental context, social influences, professional role and identity, behavioural regulation and reinforcement were found to be strong influences in both patient and staff groups
**VIEWS OF HEALTHCARE PROFESSIONALS**									
De Clercq *et al.* (2020)^37^	Inappropriate antibiotic allergy documentation in health records: a qualitative study on family physicians’ and pharmacists’ experiences	The Netherlands	A qualitative study conducted with family physicians and pharmacists based in different cooperatives (groups of family physicians) in the Netherlands	44 clinicians (34 family physicians and 10 pharmacists) from different regional groups, which use different computer software for medical records; 18 female, 26 male; mean age 44 years (age range 27–67 years); average work experience 14.5 years (range 0.5–33 years)		Focus groups	Inductive content analysis	To explore the experiences of family physicians and pharmacists performing and encountering antibiotic allergy documentation; to identify the main determinants leading to inappropriate documentation using a qualitative approach	Three themes emerged: magnitude and awareness of the problem of inappropriate antibiotic allergy documentation; origin of the problem; and approaches for addressing the problem. Participants noted that the magnitude of contamination of medical files with inappropriate documentation leads to scepticism about current documentation. Major hindering factors were electronic health record systems and electronic communication Family physicians and pharmacists believed they had insufficient knowledge about antibiotic allergies and called for tools to rectify inappropriate allergy documentation and facilitate proper documentation
Hanssen *et al.* (2021)^38^	Implementing a new antibiotic allergy protocol in clinical practice: well-trusted but not used	The Netherlands	A qualitative study of implementation of a new antibiotic allergy protocol in Internal Medicine, Pulmonary Care and Surgery inpatient wards in a Dutch hospital .	Interviews: 13 medical professionals (5 doctors and 1 nurse in Internal Medicine; 5 doctors in Pulmonary Care; 2 doctors in Surgery department). Focus groups: (i) 30 medical professionals from Internal Medicine; (ii) 5 medical professionals from Pulmonary Care .		Semi-structured interviews and focus groups	Unspecified	To study the implementation process of the new antibiotic allergy protocol	Dissemination of the protocol via the regular online hospital-wide guidance system did not have a significant impact on the knowledge about or use of the protocol. If healthcare professionals found the protocol, they thought it was valuable and expressed trust in the expertise embodied in it. Protocol use in practice was minimal Interviewees doubted the accuracy of the patients’ histories about their previous adverse drug reactions, and/or the information in their medical records and concluded that adherence to the expert guideline was needlessly risky. They felt the acute allergic reaction risk for a patient outweighed the risk of suboptimal therapy or future antimicrobial resistance
Powell *et al.* (2021)^26^	Focus group study exploring the issues and the solutions to incorrect penicillin allergy-labelled patients: an antibiotic stewardship patient safety initiative	UK	A qualitative study conducted with staff in a general district hospital without access to allergy service in England	17: 9 doctors (4 consultants, 1 consultant medical microbiologist, 3 foundation year doctors, 1 specialist trainee doctor), 4 nurses and 4 pharmacists		Focus groups	Thematic analysis, inductive approach	To explore barriers and enablers towards identifying and delabelling inpatients incorrectly labelled as penicillin allergic	Delabelling patients with incorrect penicillin allergy labels was recognized as a complex problem. Thematic analysis identified four main themes: managing penicillin-allergic patients; environmental barriers; education for patients and staff; and a future delabelling process
Sijbom *et al.* (2022)^39^	Cues to improve antibiotic-allergy registration: a mixed-method study	The Netherlands	A qualitative study conducted as part of mixed-methods evaluation of allergy registration with primary care and hospital-based healthcare professionals in the Leiden and the Hague regions of the Netherlands	34 (10 primary care physicians, 1 surgical trainee, 1 hospital physician, 2 gastroenterologists, 11 elderly care doctors, 5 elderly care nurses, and 4 pharmacists); 56% female and 53% had more than 10 years experience		Semi-structured interviews	Framework analysis	To analyse the quality of allergy registrations in primary care and to identify determinants related to the quality of registration in all involved healthcare domains	Healthcare providers’ lack of knowledge, patient factors, professional interactions, incentives and resources, capacity for organizational change, and social, political and legal factors were determinants for incorrect antibiotic allergy registration
Alagoz *et al.* (2023)^34^	Barriers to penicillin allergy delabelling in the inpatient and outpatient setting: a qualitative study	USA	A qualitative study evaluating a clinical tool for inpatient penicillin allergy delabelling in a single site veterans’ hospital in the midwestern USA	20: 3 hospitalists, 5 inpatient pharmacists, 1 infectious disease physician, 2 antimicrobial stewardship pharmacists, 4 primary care providers, 2 outpatient pharmacists, 2 resident physicians, and 1 nurse case manager for the allergy service		Semi-structured interviews	Thematic analysis, inductive approach	To describe barriers to implementing a risk-based penicillin delabelling protocol within a single site veterans’ hospital	The factors related to knowledge, skills, beliefs about capabilities, beliefs about consequences, environmental context and resources, and professional role and identity were the most prominent barriers to penicillin allergy evaluation. Heavy workload, competing priorities, and ease of access to alternative antibiotics prevented the prioritization of tasks related to delabelling. Space limitations and nursing staff shortages added to challenges in outpatient setting
Gray *et al.* (2023)^43^	Qualitative analysis of healthcare provider perspectives to evaluating beta-lactam allergies	USA, Canada, Europe	A qualitative study targeting the behaviour of evaluating the legitimacy of penicillin allergies listed in electronic medical records by healthcare professionals based in North America and Europe	25 participants (9 physicians, 9 pharmacists, 7 nurses)		Semi-structured interviews	Thematic analysis, inductive and deductive approaches	To identify barriers hindering β-lactam allergy evaluation and delabelling in practice and develop intervention designed to overcome the reported barriers	Environmental context and resources were believed to be the biggest barriers, whilst optimism, goals and emotion had least impact on β-lactam allergy evaluation and delabelling. The authors proposed development and dissemination of a clear policy for evaluation of β-lactam allergies and rework of electronic health records for documenting and utilizing allergy intervention as interventions to overcome the barriers hindering β-lactam allergy evaluation and delabelling
Alqahtani *et al.* (2024)^42^	Drug allergy management in Egypt, Sri Lanka and the Caribbean: a qualitative study	Egypt, Sri Lanka, Barbados, and Trinidad and Tobago	A qualitative study of drug allergy management in public and private heath sectors in Egypt, Sri Lanka, Barbados, and Trinidad and Tobago	90 clinicians (45 physicians, 19 pharmacists, 26 nurses); 57 females, 33 males		Virtual focus groups	Thematic analysis, inductive phenomenological approach	To gain insights into drug allergy management pathways as well as the views and perspectives of healthcare professionals regarding drug allergies in Sri Lanka, Barbados, Trinidad and Tobago, and Egypt	Egypt, Sri Lanka, Barbados, and Trinidad and Tobago lack equitable drug allergy management pathways. Computerized decision support systems alongside basic training and education might facilitate accurate labelling and delabelling
Powell *et al.* (2024)^30^	Non-allergist healthcare worker views on delivering a penicillin allergy de-labelling inpatient pathway: identifying the barriers and enablers	UK	A qualitative study of managing patients with penicillin allergy records and delivery of penicillin allergy delabelling inpatient pathway in a district general hospital in the UK	23 clinicians (11 doctors, 5 pharmacists, 2 medicines optimization technicians, 5 nurses); 14 females, 9 males		Semi-structured interviews, on-line	Thematic analysis, inductive approach	To explore the perspectives of healthcare workers in medical specialties on managing patients with penicillin allergy records and delivering a proposed penicillin allergy delabelling inpatient pathway	Penicillin allergy delabelling pathway was considered a shared responsibility of the multidisciplinary team, which needed to be structured and supported by a framework. Penicillin allergy delabelling aligns with healthcare professionals’ roles but time to deliver delabelling was a barrier. Training on the benefits of and delivery of delabelling for patients who may benefit from penicillin therapy during the current episode of care would motivate healthcare workers to deliver delabelling
Bjorbak Alnaes *et al.* (2024)^41^	‘What if the patient has a severe reaction, and it is my fault?’ A qualitative study exploring factors for sustainable implementation of penicillin allergy delabelling	Norway	A qualitative study of managing patients with penicillin allergy and sustainable implementation of a clinical pathway for penicillin allergy delabelling with nurses and physicians based in four hospitals in the Western Norway Health Region	25 (12 nurses, 13 physicians); 15 females, 10 males; 3 nurses with ≤5 years of practice; 9 nurses with ≥5 years of practice; 3 residents and 10 board certified specialist doctors		Semi-structured interviews and focus groups	Systematic text condensation analysis for cross-case, thematic analysis	To explore how penicillin allergy is perceived and identify needs to be met for the sustainable implementation of clinical pathway for penicillin allergy delabelling in Norway	Three main needs for implementation of penicillin allergy delabelling are: creating psychological safety; utilising clinicians’ inherent motivation to aid implementation, and providing optimal organizational structures
Carter et al (2024)^35^	Multifaceted implementation strategy to improve the evaluation of penicillin allergies in perioperative patients: a pre-post feasibility implementation study	USA	A qualitative study conducted as part of a pre-post feasibility implementation study of penicillin allergy evaluation in the outpatient surgical areas in an academic medical centre in the northeast USA	7 nurses		Focus group	Thematic analysis; Theoretical Framework of Acceptability	To evaluate the feasibility of the implementation strategy to improve perioperative nurses’ documentation of penicillin allergy and notifying prescribers of patients with low-risk symptoms of penicillin allergy	Whereas nurses perceived the intervention for documentation of penicillin allergy history as acceptable, nurses’ notification to prescribers of low-risk penicillin allergies had poor acceptability. Nurses expressed a lack of confidence in identifying patients with low-risk penicillin allergies and perceived the risk-stratification of penicillin allergies to be a prescribing clinician, not nursing responsibility

### Findings

**Figure 2.**
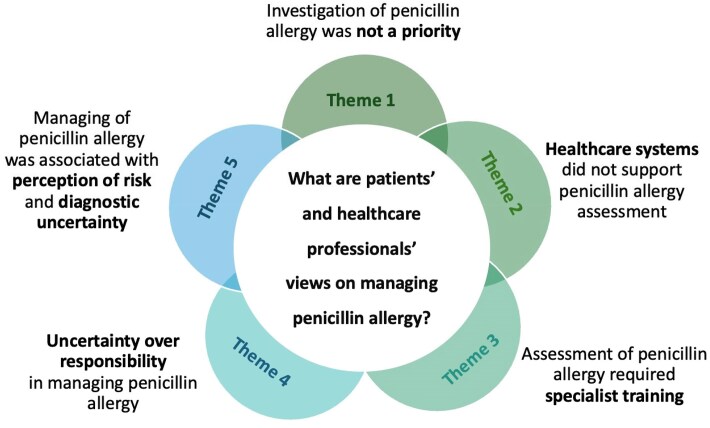
Analytical themes identified through thematic synthesis of patients’ and healthcare professionals’ views of managing penicillin allergy.

Five interconnected analytical themes were identified through thematic synthesis of patients’ and healthcare professionals’ views, which are presented in Figure 2: Theme 1—investigation of penicillin allergy was not a priority; Theme 2—healthcare systems did not support penicillin allergy assessment; Theme 3—penicillin allergy assessment required specific training; Theme 4—uncertainty over responsibility in managing penicillin allergy; and Theme 5—managing penicillin allergy was associated with perception of risk and diagnostic uncertainty.

#### Theme 1—investigation of penicillin allergy was not a priority

‘*My goal is to treat the patient immediately*’ admitted doctors reflecting on current practice in Gray *et al*.^43^ According to doctors, in both primary care and hospital, instead of assessing penicillin allergy, they opted for alternative antibiotics to expedite the initiation of treatment for infection,^25,26,30,32,34,36,37,39,43^ compromising on the long-term risks, such as treatment failure or antimicrobial resistance.^29,30,36–39^ In Wanat *et al.*,^25^ GPs justified their preference to prescribe alternative antibiotics over investigating penicillin allergy records as adherence to guidelines:

*[it] won’t be that complicated. There’s always options B. As I said, it’s actually from our local guidance that gave us that option. It’s all very well established. It’s not complicated to find an option.* ^25^

In Alagoz *et al.*,^34^ hospital doctors intended to investigate penicillin allergy when it *‘directly affected patient’s hospital course’,* whereas in Wanat *et al.*^25^ GPs reported assessing allergy histories when antibiotic options were limited or a patient reported multiple antibiotic allergies.

Echoing the views of doctors, in Carter *et al.*^31^ and Antoon *et al*.,^33^ parents were more likely to consider penicillin allergy assessment when alternative antibiotics were unavailable:

*Again, if my kid had a life-threatening bacterial infection and the only thing that was going to help them was a penicillin, yeah, I’m going to give that a shot. But if there are other things available, I’m going to try that ﬁrst*.^33^

In Carter *et al.*,^31^ parents based their preference of giving non-penicillin antibiotics to their children *‘on the notion of do no harm’* to avoid causing discomfort arising during allergy assessment. In other studies, patients either viewed penicillin allergy assessment as a low priority amongst other healthcare needs because they tolerated non-penicillin antibiotics^28^ or expressed personal beliefs that discouraged them from having an allergy assessment, e.g. there was a hereditary or a racial predisposition to penicillin allergy.^25,28,31,33,36,37,40,42^

#### Theme 2—healthcare systems did not support penicillin allergy assessment

Healthcare professionals thought that electronic health records directly hindered penicillin allergy assessment. This essential workplace-based technology, through its evolution into digital format and by design, made it difficult for healthcare professionals to record an allergy history, accurately distinguish between an allergy and side effects, identify patients with penicillin allergy, or obtain the details of previous allergic reactions.^30,34,38^ In De Clerq *et al*.,^37^ a family doctor reflected:

*When I started, we wrote an allergy at the top of ‘the green patient chart’. After the automation, there were memos, and now there is a specific field in the electronic medical files intended for intolerances, contraindications, and allergies. As a result, many registrations are mixed up.*

Another barrier to penicillin allergy assessment was created by a variable provision of specialist services reported by healthcare professionals from low-, middle- and high-income countries.^24,27,29,36,39,42^ Due to the limited access to allergy specialists, GPs who wished to investigate penicillin allergy in Sijbom *et al.*,^39^  *‘felt stuck’* because *‘these allergists have long waiting lists, though I don’t know how long. So, if you need acute assistance, that doesn’t help.’*

Although some organizations had policies for treating, reporting and reviewing allergic reactions, they were implemented to protect patients with penicillin allergy from an accidental re-exposure to penicillin rather than support healthcare professionals in assessing penicillin allergy records.^42^ Thus, the implementation of penicillin allergy assessment required an investment in solutions based on both human resources and healthcare systems, such as a dedicated organizational policy, an improvement in the quality of allergy records, and communication of allergy records between primary and secondary care.^24,26,27,29,30,32,34,37–39,41–43^

#### Theme 3—assessment of penicillin allergy required specific training

Despite feeling confident in treating anaphylaxis, doctors thought that they required different skills to assess penicillin allergy records.^30,34^ Specifically, interpreting an allergy history and distinguishing between different adverse reactions were viewed as challenging by doctors and pharmacists.^37,39^ In Sijbom *et al.*,^39^ pharmacists reflected that if they were *‘better educated, we would be better at registering it’*, and doctors noted *‘when do you call something an allergy and when a side effect? Urticaria is both a side effect and an allergy. Both are plausible, so how do I choose?’*

Going beyond the recognition of their own training needs, healthcare professionals acknowledged that the adoption of penicillin allergy assessment required a change in practice^34,36,41^ and development of a new habit of questioning rather than accepting the accuracy of penicillin allergy records:

*‘I think recognition is probably the biggest thing. It hasn’t been part of my workflow in the past to look for penicillin allergy and then to think to assess whether it’s real.’* ^34^

#### Theme 4—uncertainty over responsibility in assessing penicillin allergy

The debate over the responsibility in assessing penicillin allergy arose due to a variation in views amongst healthcare professionals on whether the responsibility should be shared or held by a particular profession. A pharmacist in Powell *et al.*^30^ noted:

*To make this pathway just be owned solely by one profession is not a very efficient use of it. So, it’s part of the making ‘every patient contact count’. Anyone involved in healthcare is involved in the allergy side of things, so you can’t be involved in healthcare and not pay attention to their allergy status.*

Healthcare professionals, who were in favour of a collaborative approach, stressed the importance of clearly defined responsibilities for staff conducting penicillin allergy assessment.^25,29,30,34,43^

Doctors viewed nurses’ role as important in taking an allergy history;^26,30,43^ however, besides the administration of drugs and performing observations, nurses felt that the evaluation of penicillin allergy history and the decision regarding patients’ suitability for penicillin allergy testing fell outside the remit of their role.^29,30,35,41,43^

Like nurses, pharmacy technicians believed that allergy history taking and documentation, but not the evaluation of allergy history, fell within the scope of their practice.^30,39^ Whilst hospital pharmacists raised concerns about capacity,^34,43^ pharmacists specializing in infectious diseases felt *‘very good in assessing allergies and investigating further’.*^43^ Hospital doctors and nurses considered pharmacists to be well prepared to assess patients with penicillin allergy records.^30,43^

Doctors felt confident in assessing patients with penicillin allergy records following a pathway but preferred to have a senior or an allergy specialist to refer to because of the perceived consequences of the decision they were making.^26,29,43^ As a hospital doctor in Ngassa *et al.*^36^ explained *‘I mean, it’s a big deal…I want to make sure this person, that they really aren’t allergic to penicillin, and who am I to make that determination.’*

Whilst some GPs were unaware of what penicillin allergy assessment entailed,^25,34^ others in Wanat *et al.*^25^ reported having experience of *‘successfully challenging allergy record previously’.*

In contrast to healthcare professionals’ views concerning their role in penicillin allergy assessment, patients trusted healthcare professionals in all aspects of managing penicillin allergy,^24–29,31–33,36,37,40^ but preferred if their own GP or paediatrician, a *‘gatekeeper’*, offered them to undergo allergy assessment.^33^

#### Theme 5—managing of penicillin allergy was associated with perception of risk and diagnostic uncertainty

Due to the fears of causing an adverse reaction,^25,29,30,32,34,36,40,41,43^ healthcare professionals reported taking additional care and consideration when treating patients with penicillin allergy records.^39,41,42^ Patients also took preventative actions and tried to mitigate the risk of accidental exposure to penicillin by reminding healthcare professionals of their allergy status to ensure it was not overlooked.^31,33,40^

Both hospital and primary care doctors felt cautious about penicillin allergy assessment and expressed the fear of litigation if a patient was harmed during assessment.^25,30,34,36,43^ In Wanat *et at.*,^25^ a GP described it as *‘kind of indefensible, isn’t it really’* if they independently challenged a documented penicillin allergy and the patient developed an adverse reaction.

Patients expressed their own concerns about feeling discomfort during allergy assessment^23,24,28,31,33,40^ and wanted a penicillin allergy test that was safe, well explained and provided an accurate result.^24,27–29,33,36,40^ Patients were concerned about feeling well enough to fully understand the risks and benefits of penicillin allergy evaluation, wanted to have sufficient time to deliberate whether they should undertake the assessment, and to have time to adjust to the renewed allergy status after completing the test.^23,27,28,31–33,39,40,44^

Although most patients and healthcare professionals agreed that a negative allergy test successfully excluded penicillin allergy,^24,27,33,36,40^ some remained concerned about the recurrence of adverse reactions to penicillin in future.^24,25,29,33–36,40,43^ A patient after a negative penicillin allergy test in Wanat *et al.*^24^ described a lingering concern:

*Cause I’ve lived with that fear, if anybody gives me penicillin, I’m gonna die sort of thing, for years you know, from being a baby so of course you can’t just terminate a fear like that. It’s still there in the back of your mind all the time.*

## Discussion

### Summary of findings

Using thematic synthesis, we interpreted the findings of 21 studies that reported patients’ and healthcare professionals’ views on managing penicillin allergy. Neither patients nor healthcare professionals viewed penicillin allergy assessment as a priority in routine care, but both acknowledged its value when antibiotic options were limited.

From healthcare professionals’ perspective, existing healthcare systems hindered penicillin allergy assessment. A perceived need for specialist training and uncertainty over professional responsibility in penicillin allergy assessment influenced how healthcare professionals managed patients with penicillin allergy records. Both patients and healthcare professionals associated managing penicillin allergy with risk due to fears of recurrent reactions to penicillin during allergy testing, and some expressed uncertainty over subsequent use of penicillin despite a negative allergy test.

### Comparison with existing literature

Despite the emphasis in the global antimicrobial stewardship strategy on the importance of penicillin allergy assessment,^12^ in our review we found that patients and healthcare professionals did not view the investigation of penicillin allergy records as a priority, suggesting that the public health message of the benefits of clarifying penicillin allergy status has not yet translated into routine clinical practice or reached the public.

We found that workplace-based systems directly influenced how healthcare professionals managed patients with penicillin allergy records. Similar to a narrative review by Jani *et al.*,^17^ our review highlighted the importance of understanding systemic capabilities relevant to the adoption of penicillin allergy assessment interventions in a healthcare setting.

Healthcare professionals’ view that specific training was required to assess patients’ penicillin allergy status identified in our review is consistent with previous studies that identified knowledge gaps related to managing antibiotic allergies amongst non-allergy specialists.^45,46^

We found that healthcare professionals expressed varied opinions on having professional responsibility for assessing patients’ penicillin allergy status. Although assessment of patients with a low-risk penicillin allergy history is typically delivered by multidisciplinary teams, the evidence supporting penicillin allergy assessment programmes led by a single profession is growing.^47,48^ Chow *et al.*^49^ also demonstrated that non-allergists could safely assess patients using skin testing, creating opportunities for patients with more complex allergy histories to be assessed and tested outside specialist setting.

In our review both patients and healthcare professionals associated managing penicillin allergy with risk. The perception of risk associated with managing penicillin allergy could reflect patients’ and healthcare professionals’ awareness of medication errors with penicillin occurring in the real world and resulting in patient harm and litigation.^50^ In a systematic review of safety of penicillin allergy testing, Loprete *et al.*^51^ found that anaphylaxis occurred in 0.3% (95/98 316) of participants recruited in 95 penicillin allergy delabelling studies, indicating that penicillin allergy testing should not be considered risk free. However, intentional assessment of penicillin allergy records, through systematic evaluation of allergy history undertaken with a patient’s consent and followed by testing where appropriate, is a process of risk mitigation. In an ethical analysis, Xiang *et al.*^52^ argued that for patients, the benefits of being able to have penicillin antibiotics due to a renewed allergy status outweighed the risk of harms caused by using second-line antibiotics.

### Implications for practice

Our finding that neither patients nor healthcare professionals viewed penicillin allergy assessment as a priority suggests that there is a need to review how information about the negative consequences of penicillin allergies and the need for penicillin allergy assessments is communicated. Clearer definition of healthcare professionals’ roles and responsibilities and leadership accountable for the development of policies, training and implementation strategies for penicillin allergy assessment are needed. However, given the complexity of penicillin allergy assessment and the presence of competing clinical priorities, consideration should be given to whether establishing a designated clinical service as opposed to implementing pathways aiming to change the existing clinical practice would create more opportunities for patients with penicillin allergy records to be assessed.

The impact of guidelines on prioritization of penicillin allergy assessment should not be overlooked. Where penicillin antibiotics should be used first, guidelines should mandate the assessment of penicillin allergy history and allergy testing prior to use of second-line antibiotics. However, there is evidence that guidelines alone will not change behaviours.

We also identified that changes in workplace-based tools were needed to help healthcare professionals assess penicillin allergy records. Thus, a redesign of digital health records with improved features for documentation of allergy history could facilitate penicillin allergy assessment.

Considering the perceived need for specialist training to assess penicillin allergy identified in our review, the knowledge gaps in managing penicillin allergy amongst healthcare professions should be addressed strategically and systematically, starting with undergraduate training and including qualified healthcare professionals. Provision of education of patients regarding the benefits of penicillin allergy assessment is also needed to address existing misconceptions discouraging them from allergy testing.

Healthcare professionals’ uncertainty over responsibility in managing penicillin allergy found in our review could hinder the adoption of penicillin allergy assessment pathways. Perception of risk associated with managing penicillin allergy found in our review could discourage patients and healthcare professionals from assessing penicillin allergy records or using penicillin antibiotics after a negative allergy test. Information on safety of penicillin allergy assessment and use of penicillin following a negative allergy should be included in the educational materials to alleviate patients’ and healthcare professionals’ concerns. Further research exploring the balance of risks associated with managing penicillin allergy, including the risk of assessing and testing penicillin allergy, the risk of recurrent reaction to penicillin, and the risk of not testing, is needed.

The implications for managing penicillin allergy based on the findings of our review are summarized in Table 3.

**Table 3.** Summary of implications for managing penicillin allergy based on the review findings

Findings	Implications
Investigation of penicillin allergy was not a priority	Education of patients and healthcare professionals about negative consequences of penicillin allergy labels Education of patients and healthcare professionals about penicillin allergy assessment and testing processes
Healthcare systems did not support penicillin allergy assessment	Redesign of electronic allergy records to improve documentation of allergy history and severity of the reactions Implementation of penicillin allergy assessment across different healthcare sectors focusing on provision of guidelines with pathways for testing locally or referring for testing to a specialist Investment in designated penicillin allergy assessment processes/procedures for low-risk patients outside specialist setting
Assessment of penicillin allergy required specific training	Development of national curricula in allergy for undergraduate and graduate training programmes for all healthcare professionals Training for all qualified healthcare professionals ensuring multidisciplinary teams have necessary skills to assess penicillin allergy records
Uncertainty over responsibility in managing penicillin allergy	Multidisciplinary approach to penicillin allergy assessment with clear allocation of tasks Division of roles and responsibilities with support from specialists
Managing penicillin allergy was associated with perception of risk and diagnostic uncertainty	Penicillin allergy history assessment using risk stratification to identify patients suitable for allergy testing Provision of information of risks of adverse reactions during penicillin allergy testing Education on the incidence of true allergy and distinction between allergy and non-immune-mediated side effects

### Strengths and limitations

To our knowledge, this is the first qualitative evidence synthesis of the published literature pertaining to patients’ and healthcare professionals’ views on managing penicillin allergy. The findings of this review offer a novel interpretation and overview of the evidence and further the understanding of contextual factors and influences that impact the care of patients with penicillin allergy records in clinical practice.

However, all studies, except one, presented the perspectives of patients and healthcare professionals from Europe and North America. We also identified fewer studies from primary care and only one study from low- or middle-income countries, which may limit transferability of findings of our review outside the hospital setting in high-income countries.

More studies reported findings of healthcare professionals’ than patients’ views. Despite searching for studies with any group of healthcare professionals, we found no studies that reported allergy specialists’ views and met the inclusion criteria, and thus the findings of this review reflect non-allergists’ views on managing penicillin allergy.

Whilst the implementation of penicillin allergy assessment in a specific occupational context will be influenced by healthcare professionals’ views, determinants other than professional role may be relevant. Due to mixed reporting of gender and age and absence of reporting of ethnicity and deprivation data in the primary studies, we were unable to analyse how patients’ or healthcare professionals’ views on managing penicillin allergy varied across different gender, socioeconomic and ethnic groups.

### Conclusions

We found that both patients and healthcare professionals associated managing penicillin allergy with risk, and neither viewed penicillin allergy assessment as a priority in routine care.

Based on the findings of our review, we propose a provision of structured education for patients and healthcare professionals emphasizing the benefits of penicillin allergy assessment and explaining the known risks of allergy assessment.

To embed penicillin allergy assessment into routine practice, we suggest a revision in guidelines to mandate penicillin allergy assessment prior to initiation of the second-line antibiotics and a redesign of electronic health records to improve allergy documentation and assessment.

Considering the limited access to the specialist allergy services and the need to assess patients with a documented penicillin allergy, research should continue to explore new models for penicillin allergy assessment services involving different healthcare settings.

There is a need to engage people with a broad range of characteristics in research to ensure the representation of diverse populations and understand their views relevant to managing penicillin allergy.

## Supplementary Material

dlag049_Supplementary_Data

## Data Availability

All data included in the findings of this qualitative evidence synthesis are available from the previously published articles cited in this manuscript.

## Transparency declarations

K.K. has nothing to declare. M.W. is supported by the National Institute for Health and Care Research (NIHR) Health Protection Research Unit in Healthcare Associated Infections and Antimicrobial Resistance (NIHR207397), a partnership between the UK Health Security Agency (UKHSA) and the University of Oxford. The views expressed are those of the author and not necessarily those of the NIHR, UKHSA, or the Department of Health and Social Care. N.W.R. has nothing to declare. J.S. is co-lead for the British Society for Antimicrobial Chemotherapy massive online open access penicillin allergy course; is a member of the British Society for Allergy and Clinical immunology penicillin allergy working party, the European Society for Clinical Microbiology and Infectious Diseases antibiotic allergy guideline group, and the Department of Health antibiotic prescribing, resistance and healthcare associated infection group. He has received research funding from the Medical Research Council, the Wellcome Trust, the NIHR, Engineering and Physical Sciences Research Council and Lumos diagnostics. He is supported in part by the NIHR Leeds Biomedical Research Centre (NIHR203331). S.T.-C. is supported by the NIHR Health Protection Research Unit in Healthcare Associated Infections and Antimicrobial Resistance (NIHR207397), a partnership between the UKHSA and the University of Oxford. The views expressed are those of the authors and not necessarily those of the NIHR, UKHSA or the Department of Health and Social Care.

## Supplementary data

Tables S1–S5 are available as Supplementary data at *JAC-AMR* Online.
