# Supplementary material for: What are patients’ and healthcare professionals’ views on managing penicillin allergy? A qualitative evidence synthesis
Source: JAC Antimicrob Resist. 2026 Apr 20;8(2):dlag049. doi: 10.1093/jacamr/dlag049 (PMC13092344; doi:10.1093/jacamr/dlag049)
Supplement: dlag049_Supplementary_Data [file dlag049_supplementary_data.docx]

# Supplementary data

Table S1: ENTREQ reporting checklist

| **No** | **Item** | **Guide and description** | **Reported on page** |
| --- | --- | --- | --- |
| 1 | Aim | State the research question the synthesis addresses. | Title, 5 |
| 2 | Synthesis methodology | Identify the synthesis methodology or theoretical framework which underpins the synthesis, and describe the rationale for choice of methodology (e.g. meta-  ethnography, thematic synthesis, critical interpretive synthesis, grounded theory synthesis, realist synthesis, meta-aggregation, meta-study, framework synthesis). | 5, 8 |
| 3 | Approach to searching | Indicate whether the search was pre-planned (comprehensive search strategies to seek all available studies) or iterative (to seek all available concepts until theoretical saturation is achieved). | 7, 8 |
| 4 | Inclusion criteria | Specify the inclusion/exclusion criteria (e.g. in terms of population, language, year limits, type of publication, study type). | 8 |
| 5 | Data sources | Describe the information sources used (e.g. electronic databases (MEDLINE, EMBASE, CINAHL, psychINFO, Econlit), grey literature databases (digital thesis, policy reports), relevant organisational websites, experts, information specialists, generic web searches (Google Scholar), hand searching, reference lists) and when the searches were conducted; provide the rationale for using the data sources. | 7, 9 |
| 6 | Electronic  Search  strategy | Describe the literature search (e.g. provide electronic search strategies with population terms, clinical or health topic terms, experiential or social phenomena  related terms, filters for qualitative research and search limits). | Supplementary material Table S2 |
| 7 | Study  screening  methods | Describe the process of study screening and sifting (e.g. title, abstract and full text review, number of independent reviewers who screened studies). | 8 & Figure 1 |
| 8 | Study  characteristics | Present the characteristics of the included studies (e.g. year of publication, country, population, number of participants, data collection, methodology, analysis, research questions). | 9, 10 & Table 2 |
| 9 | Study selection results | Identify the number of studies screened and provide reasons for study exclusion (e.g. for comprehensive searching, provide numbers of studies screened and reasons for exclusion indicated in a figure/flowchart; for iterative searching describe reasons for study exclusion  and inclusion based on modifications the research question and/or contribution to theory development). | 9, Figure 1, supplementary data Table S3 |
| 10 | Rationale for  appraisal | Describe the rationale and approach used to appraise the included studies or selected findings (e.g. assessment of conduct (validity and robustness), assessment of reporting (transparency), assessment of content and utility of the findings). | 8 |
| 11 | Appraisal items | State the tools, frameworks and criteria used to  appraise the studies or selected findings (e.g. Existing tools: CASP, QARI, COREQ, Mays and Pope [25]; reviewer developed tools; describe the domains assessed: research team, study design, data analysis and interpretations, reporting). | 8 |
| 12 | Appraisal  process | Indicate whether the appraisal was conducted  independently by more than one reviewer and if  consensus was required. | 8 |
| 13 | Appraisal results | Present results of the quality assessment and indicate which articles, if any, were weighted/excluded based on the assessment and give the rationale. | 9 & Supplementary data Table S3 |
| 14 | Data extraction | Indicate which sections of the primary studies were analysed and how were the data extracted from the primary studies? (e.g. all text under the headings “results /conclusions” were extracted electronically and entered into a computer software). | 8 |
| 15 | Software | State the computer software used, if any. | 8 |
| 16 | Number of  reviewers | Identify who was involved in coding and analysis. | 8, 9 |
| 17 | Coding | Describe the process for coding of data (e.g. line by line coding to search for concepts). | 8, 9 |
| 18 | Study  comparison | Describe how were comparisons made within and across studies (e.g. subsequent studies were coded into pre-existing concepts, and new concepts were created when deemed necessary). | 8, 9 |
| 19 | Derivation of  themes | Explain whether the process of deriving the themes or constructs was inductive or deductive. | 8, 9, supplementary data Table S4 and Table S5. |
| 20 | Quotations | Provide quotations from the primary studies to illustrate themes/constructs, and identify whether the quotations were participant quotations or the author’s interpretation | 20 - 25 |
| 21 | Synthesis  output | Present rich, compelling and useful results that go beyond a summary of the primary studies (e.g. new interpretation, models of evidence, conceptual models, analytical framework, development of a new theory or construct). | 20 – 25, 26, 27, Figure 2, Table 3, |

Table S2: Search strategy for Medline database

|  | [Medline (Ovid MEDLINE® Epub Ahead of Print, In-Process & Other Non-Indexed Citations, Ovid MEDLINE® Daily and Ovid MEDLINE®) 1946 to present](https://ovidsp.ovid.com/ovidweb.cgi?T=JS&NEWS=N&PAGE=main&SHAREDSEARCHID=2dkZGAqzPYCABImGYekjaSqtsE2QSXMNFeLSfRSnE6utwoH9omuRn2ktRYvIsGBWS) |
| --- | --- |
| 1 | exp Penicillins/ |
| 2 | beta-Lactams/ |
| 3 | Anti-Bacterial Agents/ |
| 4 | (penicillin? or amoxicillin or ampicillin or amdinocillin or co-amoxiclav or cyclacillin or flucloxacillin or methicillin or nafcillin or oxacillin or phenoxymethylpenicillin or sulbactam or ticarcillin).ti,kf. |
| 5 | (antibiotic? or anti-biotic? or antibacterial? or anti-bacterial? or antimicrobial? or anti-microbial? or b-lactam? or beta lactam?).ti,kf. |
| 6 | 1 or 2 or 3 or 4 or 5 |
| 7 | Drug hypersensitivity/ |
| 8 | hypersensitivity/ or hypersensitivity, immediate/ or anaphylaxis/ |
| 9 | Drug-Related Side Effects and Adverse Reactions/ |
| 10 | (allerg* or sensitiv* or hypersensitiv* or anaphyl* or intoleran* or toleran*).ti,kf. |
| 11 | ((adverse adj2 (effect? or reaction? or event? or outcome?)) or adrs).ti,kf. |
| 12 | 7 or 8 or 9 or 10 or 11 |
| 13 | 6 and 12 |
| 14 | exp Penicillins/ae |
| 15 | beta-Lactams/ae or Anti-Bacterial Agents/ae |
| 16 | ((penicillin? or amoxicillin or ampicillin or amdinocillin or co-amoxiclav or cyclacillin or flucloxacillin or methicillin or nafcillin or oxacillin or phenoxymethylpenicillin or sulbactam or ticarcillin) adj5 (allerg* or sensitiv* or hypersensitiv* or anaphyl* or intoleran* or toleran*)).ab. |
| 17 | ((penicillin? or amoxicillin or ampicillin or amdinocillin or co-amoxiclav or cyclacillin or flucloxacillin or methicillin or nafcillin or oxacillin or phenoxymethylpenicillin or sulbactam or ticarcillin) and (adverse effect? or adverse reaction? or adverse event? or adverse outcome? or adverse drug effect? or adverse drug reaction? or adverse drug event? or adverse drug outcome? or adrs)).ab. |
| 18 | 13 or 14 or 15 or 16 or 17 |
| 19 | grounded theory/ or exp qualitative research/ |
| 20 | focus groups/ or interviews as topic/ |
| 21 | ("Attitude of Health Personnel"/ or attitude to health/ or health knowledge, attitudes, practice/ or "patient acceptance of health care"/ or exp patient satisfaction/) and (survey? or questionnaire?).mp. |
| 22 | qualitative.ti,kf. or (qualitative adj3 (stud* or data or finding? or evaluation or analysis)).ab. |
| 23 | (interview* or focus group* or ((unstructured or semi-structured or semistructured or open) adj2 question*) or case stud*).ti,ab,kf. |
| 24 | (grounded theory or ethnograph* or phenomenolog* or ipa or narratives or narrative analysis or themes or thematic analysis).ti,ab,kf. |
| 25 | (mixed method* or multi* method* or multimethod* or mixed stud*).ti,ab,kf. |
| 26 | ((qualitative adj2 review) or metaethnograph* or meta-ethnograph* or metasynthes* or meta-synthes*).ti,ab,kf. |
| 27 | 19 or 20 or 21 or 22 or 23 or 24 or 25 or 26 |
| 28 | 18 and 27 |
| 29 | exp animals/ not humans/ |
| 30 | 28 not 29 |

Table S3: Summary of quality assessment scores of studies using CASP checklist

| **Author (year)** | **Section A: Are the results valid?** | | | | | | **Section B: What are the results?** | | | **Section C: Will the results help locally?** | | **Score (max 18 points)** |
| --- | --- | --- | --- | --- | --- | --- | --- | --- | --- | --- | --- | --- |
|  | 1. Was there a clear statement of the aims of the research? | 2. Is qualitative methodology appropriate? | 3. Was the research design appropriate to address the aims of the research? | 4. Was the recruitment strategy appropriate to the aims of the research? | 5. Was the data collected in a way that addressed the research issue? | 6. Has the relationship between researcher and participants been adequately considered? | 7. Have ethical issues been taken into consideration? | 8. Was the data analysis sufficiently rigorous? | 9. Is there a clear statement of findings? | 10. How valuable is the research? | Yes - 2 points, Can't tell - 1 point, No - 0 |  |
| **Savic, et al. (2019)** | Yes | Yes | Can't tell | Yes | Can't tell | Can't tell | Can't tell | Can't tell | Can't tell | Unique setting - pre-operative assessment unit; mixed-methods study with a limited amount of qualitative data reported. | Satisfactory paper | 12 |
| **Wanat, et al. (2019)** | Yes | Yes | Yes | Yes | Yes | No | No | Yes | Yes | First UK based study in primary care aiming to identify barriers and enablers for penicillin allergy testing and subsequent antibiotic use. | Key paper | 14 |
| **De Clercq, et al. (2020)** | Yes | Yes | Yes | Yes | Yes | Yes | Yes | Yes | Yes | The first paper reporting on the management of penicillin allergy records and factors affecting quality of penicillin allergy records in Dutch primary care. | Valuable paper | 18 |
| **Protudjer, et al. (2020)** | Yes | Yes | Yes | Can't tell | Yes | Can't tell | Yes | No | Yes | Gives insight into parents' and children’s experiences of having been falsely labelled as allergic to penicillin and their experience of de-labelling. | Key paper | 14 |
| **Hanssen, et al. (2021)** | Yes | Yes | Yes | Yes | Yes | Can't tell | Yes | No | Yes | Reporting views on clinical decision support tool aimed to improve penicillin allergy assessment highlighting challenges at organisational level. | Valuable paper | 15 |
| **Powell, et al. (2021)** | Yes | Yes | Yes | Can't tell | Yes | Can't tell | Yes | Yes | Yes | Reporting views of healthcare professionals based in an organisation without access to specialist allergy service. | Key paper | 16 |
| **Wanat, et al. (2021)** | Yes | Yes | Yes | Yes | Yes | Can't tell | Yes | Yes | Yes | First UK study reporting on primary care physicians' and patients' views of penicillin allergy and their experiences in managing penicillin allergy in primary care. | Key paper | 17 |
| **Sijbom, et al. (2022)** | Yes | Yes | Yes | Can't tell | Yes | Can't tell | Yes | Yes | Yes | Dutch study, interviewees represent a range of healthcare professionals across different sectors; analysis following checklist by Flottorp, et al., which is different to TDF. | Valuable paper | 16 |
| **Wanat, et al. (2022)** | Yes | Yes | Yes | Yes | Yes | Yes | Can't tell | Yes | Yes | Study reporting views of primary care physicians and patients who received behaviour intervention package as part of ALABAMA study. | Key paper | 17 |
| **Alagoz, et al. (2023)** | Yes | Yes | Yes | Can't tell | Yes | No | Yes | Yes | Yes | Incorporates theory in intervention development and highlights individual and system-level barriers; includes healthcare staff based both in primary and secondary care. | Key paper | 15 |
| **Antoon, et al. (2023)** | Yes | Yes | Yes | Yes | Yes | Can't tell | Can't tell | Yes | Yes | Goes beyond thematic analysis of parental perceptions to develop a theoretical framework for decision to engage in penicillin allergy delabelling. | Key paper | 16 |
| **Carter, et al. (2023)** | Yes | Yes | Yes | Yes | Yes | Can't tell | Can't tell | Yes | Yes | Study captures the views of the parents of children with penicillin allergy which very recruited as members of public. | Key paper | 16 |
| **Gillespie, et al. (2023)** | Yes | Yes | Yes | No | Yes | No | Yes | Yes | Yes | Applies Consolidated Framework for Implementation Science Model supplemented by the Health Belief Model to identify barriers to penicillin allergy assessment at organisational level; patients recruited from psychiatry and dementia wards. | Key paper | 14 |
| **Gray, et al. (2023)** | Yes | Yes | Yes | Yes | Can't tell | Yes | Yes | Can't tell | Yes | Study offers summary of individual-level barriers to evaluating penicillin allergy based on their experiences in practice. | Key paper | 16 |
| **Alqahtani, et al. (2024)** | Yes | Yes | Yes | Can't tell | Yes | Can't tell | Yes | Yes | Yes | First study capturing perspectives of healthcare staff from lower-middle income countries (e.g. Egypt, Sri Lanka). Required inspection of supplementary materials. | Valuable paper | 16 |
| **Bjorbak Alnaes, et al. (2024)** | Yes | Yes | Yes | Yes | Yes | Yes | Yes | Yes | Yes | First study providing in depth understanding of clinicians' needs and contextual factors which affect the assessment of penicillin allergy in Norway; contextual findings could be translated outside Scandinavian healthcare setting. | Key paper | 18 |
| **Carter, et al. (2024)** | Yes | Yes | Yes | Can't tell | Can't tell | Can't tell | Yes | Can't tell | Yes | Study presents nurses', as occupational group’, perspectives on protocol for penicillin allergy documentation, evaluation of risk, and referral to prescribers. | Satisfactory paper | 14 |
| **Jani, et al. (2024)** | Yes | Yes | Yes | Yes | Yes | Yes | Yes | Yes | Yes | Study captures the views of patients who were considered to be low risk for allergy to penicillin and underwent direct oral challenge; study highlights complexities of adoption of direct oral challenge in different study sites in English NHS. | Key paper | 18 |
| **Ngassa, et al. (2024)** | Yes | Yes | Can't tell | Can't tell | Can't tell | Can't tell | Can't tell | Can't tell | Can't tell | Indicates there are racial and ethnic differences in beliefs regarding penicillin allergy. A short report. | Satisfactory paper | 11 |
| **Powell, et al. (2024)** | Yes | Yes | Yes | Can't tell | Yes | Can't tell | Yes | Yes | Yes | Highlights perspectives of non-allergy specialists based in a general district hospital in the UK. | Key paper | 16 |
| **Powell, et al (2024)** | Yes | Yes | Yes | Yes | Yes | No | Yes | Yes | Yes | Paper includes perspectives of eligible patients who both agreed and declined to undergo penicillin allergy assessment. | Key paper | 16 |

| **Analytical themes** | **Descriptive themes** |
| --- | --- |
| Investigation of penicillin allergy was not a priority | Patients and healthcare professionals saw penicillin allergy testing as important for ensuring optimal care.  Penicillin allergy assessment was not considered a priority by healthcare professionals and patients.  Preference by healthcare professionals and patients to use alternative antibiotics rather than investigate penicillin allergy.  Patients had misconceptions about penicillin allergy which discouraged them from having the assessment. |
| Healthcare systems did not support penicillin allergy assessment | Healthcare professionals followed systems and processes for reporting and responding to adverse reactions to penicillin.  Medical records used for allergy documentation hindered penicillin allergy assessment.  Healthcare professionals thought access to specialist allergy services was limited and variable.  Healthcare professionals perceived penicillin allergy assessment as resource intensive.  Healthcare professionals thought that organisational policy and better infrastructure would enable them to assess penicillin allergy. |
| Assessment of penicillin allergy required specific training | Healthcare professionals found it difficult to distinguish between allergy and side effects.  Healthcare professionals felt they required training and support to carry out penicillin allergy assessment. |
| Uncertainty over responsibility in managing penicillin allergy | Patients relied on healthcare professionals for guidance on managing penicillin allergy.  Patients wanted autonomy when it came to penicillin allergy testing and confirmation of their allergy status.  Healthcare professionals acknowledged their role in managing penicillin allergy. |
| Managing penicillin allergy was associated with perception of risk and diagnostic uncertainty | Patients and healthcare professionals felt reluctant to challenge penicillin allergy records.  Healthcare professionals took exceptional care to prevent accidental use of penicillin in patients with penicillin allergy labels.  Patients wanted a penicillin allergy test that was safe, well-explained and which provided an accurate result.  Patients and healthcare professionals remained concerned about future reactions to penicillin despite a negative allergy test. |

Table S4: Analytical themes with associated descriptive themes

Table S5: Map of study contribution to the development of analytical themes and associated descriptive themes

| Source paper | Theme 1 - Healthcare systems did not support penicillin allergy assessment | | | | | Theme 2 - Investigation of penicillin allergy was not a priority | | | | Theme 3 - Assessment of penicillin allergy required specific training | | Theme 4 - Uncertainty over responsibility in managing penicillin allergy | | | Theme 5 - Managing penicillin allergy was associated with perception of risk and diagnostic uncertainty | | | |
| --- | --- | --- | --- | --- | --- | --- | --- | --- | --- | --- | --- | --- | --- | --- | --- | --- | --- | --- |
|  | Healthcare professionals followed systems and processes for reporting and responding to adverse reactions to penicillin. | Medical records used for allergy documentation hindered penicillin allergy assessment. | Healthcare professionals thought access to specialist allergy services was limited and variable. | Healthcare professionals perceived penicillin allergy assessment as resource intensive. | Healthcare professionals believed that organisational policy and better infrastructure would enable them to assess penicillin allergy. | Patients and healthcare professionals saw penicillin allergy testing as important for ensuring optimal care. | Penicillin allergy assessment was not considered a priority by healthcare professionals and patients. | Preference by healthcare professionals and patients to use alternative antibiotics rather than investigate penicillin allergy. | Patients had misconceptions about penicillin allergy which discouraged them from having the assessment. | Healthcare professionals found it difficult to distinguish between allergy and side effects. | Healthcare professionals felt they require training and support to carry out penicillin allergy assessment. | Patients relied on healthcare professionals for guidance on managing penicillin allergy. | Healthcare professionals acknowledged their role in managing penicillin allergy. | Patients wanted autonomy when it came to penicillin allergy testing and confirmation of their allergy status. | Patients and healthcare professionals took exceptional care to prevent having/giving penicillin to patients with penicillin allergy labels. | Patients and healthcare professionals felt reluctant to challenge penicillin allergy records. | Patients wanted a penicillin allergy test that was safe, well-explained and which provided an accurate result. | Patients and healthcare professionals remained concerned about future reactions to penicillin despite a negative allergy test. |
| **Views of PATIENTS and/or PARENTS or CAREGIVERS** | | | | | | | | | | | | | | | | | | |
| Savic et al (2019) |  |  |  |  |  |  |  |  |  |  |  |  |  | x |  |  | x |  |
| Protudjer et al (2020) |  |  |  |  |  | x | x |  | x |  |  | x |  | x | x | x | x | x |
| Antoon et al (2023) |  |  |  |  |  | x | x | x | x |  |  | x |  | x | x |  | x | x |
| Carter et al (2023) |  |  |  |  |  | x | x | x | x |  |  | x |  | x | x |  | x | x |
| Powell et al (2024) |  |  |  |  |  | x | x |  | x |  |  | x |  | x |  |  | x |  |
| **Views of PATIENTS and HEALTHCARE professionals** | | | | | | | | | | | | | | | | | | |
| Wanat et al (2019) |  | x | x |  | x | x | x |  |  | x | x | x |  | x |  |  | x | x |
| Wanat et al (2021) |  | x |  |  |  | x | x | x | x | x |  | x | x | x | x | x |  |  |
| Wanat et al (2022) |  |  | x | x | x | x | x |  |  |  | x | x | x | x |  |  | x | x |
| Gillespie et al (2023) | x | x |  | x | x | x | x | x | x |  | x | x | x | x |  | x |  |  |
| Ngassa et al (2024) |  | x | x | x |  | x | x | x | x |  | x | x | x |  |  | x | x | x |
| Jani et al (2024) |  | x | x |  | x | x | x | x |  |  | x | x |  | x |  | x | x | x |
| **Views of HEALTHCARE professionals** | | | | | | | | | | | | | | | | | | |
| De Clercq et al (2020) |  | x |  | x | x |  |  | x | x | x | x | x | x |  |  |  |  |  |
| Hanssen et al (2021) |  | x |  |  | x |  | x | x |  |  |  |  |  |  |  |  |  |  |
| Powell et al (2021) |  | x |  |  | x | x | x | x | x |  | x | x | x |  |  |  |  |  |
| Sijbom et al (2022) | x | x | x | x | x |  | x | x | x | x | x |  | x | x | x |  |  |  |
| Alagoz et al (2023) |  | x |  | x | x | x | x | x |  | x | x |  | x |  |  | x |  | x |
| Gray et al (2023) |  | x |  | x | x | x | x | x |  |  | x |  | x |  |  | x |  | x |
| Alqahtani et al. (2023) | x | x | x | x | x |  | x |  | x | x |  |  |  |  | x |  |  |  |
| Powell et al (2024) | x | x |  | x | x |  | x | x |  | x | x |  | x |  |  | x |  |  |
| Bjorbak Alnæs et al (2024) |  |  |  | x | x | x |  |  |  |  | x |  | x |  | x | x |  |  |
| Carter et al (2024) |  |  |  |  |  |  | x |  |  |  |  |  | x |  |  |  |  | x |
